# Editorial: Optimizing medication management in older adults: addressing polypharmacy, deprescribing and represcribing challenges

**DOI:** 10.3389/fmed.2026.1824505

**Published:** 2026-04-08

**Authors:** Doron Garfinkel, Martin Wehling, Ian Scott

**Affiliations:** 1 Center for Appropriate Medication Use, Sheba Medical Center, Ramat Gan, Israel; 2 Medical Faculty Mannheim, University of Heidelberg, Heidelberg, Germany; 3 Queensland Digital Health Centre, University of Queensland, Brisbane, QLD, Australia

**Keywords:** de-prescribing, geriatric-palliative approach, inappropriate medication use, multi-morbidity, polypharmacy, quality of life, re-prescribing

Paradoxically, the blessed increase in average lifespan of recent generations has also resulted in an unprecedented time-related increase in the number of vulnerable subpopulations for whom we have previously suggested the acronym VOCODFLEX—very old people, those with co-morbidities, dementia, frailty and limited life expectancy. Older people who demonstrate one or several of these characteristics demonstrate increased disability and suffering that endure over prolonged periods of time prior to death (1–6). The inability of the workforce to balance the unprecedented medical, economic, and social needs of VOCODFLEX presages a huge “geriatric boom burden” and a tsunami of Inappropriate Medication Use and Polypharmacy (IMUP) in 21st century healthcare. This is driven in part by increasing numbers of specialists in different disciplines, each recommending interventions based on clinical practice guidelines developed within their field of expertise. Unfortunately, many of these guideline recommendations have little evidence-based proof of a positive benefit/risk ratio in older people. For example, Boyd et al. (7) concluded that “adhering to current guidelines in elders with co-morbidities may lead to inappropriate clinical judgment, creates perverse incentives to care, and diminishes the quality of care.” In addition, the absence of a single, assertive case manager capable of taking a holistic, goal-oriented view of a patient's care means there is no counterbalancing force to minimize inappropriate care that may not be in the patient's best interests. Families and care-givers are often relegated to a passive, “bystander” role (8) and this resultant diffusion of responsibility favors the proliferation of drugs, interactions and adverse drug events (ADE). The lack of a collaborative multi-disciplinary or integrative approach exacerbates the growth of IMUP.

The harms of IMUP are well-established and include cognitive and functional impairments, delirium, malnutrition and weight loss, falls and associated hip fractures, incontinence, hospitalizations, nursing home placement, decrease in quality of life (QoL) and indeed the quality of dying. IMUP also predisposes to non-adherence to effective medications which may actually benefit patients (9). The negative economic outcomes and financial burdens imposed on healthcare systems by IMUP are also huge, comprising escalating drug costs and expense of drug related hospitalizations. Furthermore, IMUP begets IMUP in a vicious cycle of over-diagnosis and over-treatment as adverse medication-related events are misdiagnosed as new diseases which warrant investigation and prescribing of yet more medications. These human, economic and social ramifications of IMUP justify it as being “The Iatrogenic Epidemic.” Therefore, it is important that all healthcare providers and health authorities be made more aware of this epidemic and ways to prevent or mitigate it (3, 4). We hope that the diverse compilation of contemporary studies published in this Research Topic represent an important step toward achieving this goal.

These articles include studies from undeveloped as well as highly developed countries and the topics range from studies on the rate of IMUP in a specific geographic region, through reports on specific drug related clinical syndromes to proposed interventions to combat IMUP.

As there is little reliable research to establish prescribing guidelines in older people, particularly VOCODFLEX, the medical community globally falls back on extrapolating results of randomized-controlled trials (RCT) performed in robust, much younger, healthier participants to all older people, including VOCODFLEX. These extrapolations may cause more harm than good (5–7, 10). Even ethical, knowledgeable medical doctors, while aware of the risk of IMUP, continue to prescribe inappropriately, feeling secure in the belief that they are following guidelines that rest upon the results of multiple, large RCTs. In contrast, there is a need for greater caution and appreciation of the limited generalizability of these trials and an acknowledgment that in many older people we cannot be sure we are conferring net benefit.

Similar to efforts applied to pandemics, a global coalition is needed to counter the scourge of IMUP, which led in 2009 to the establishment of IGRIMUP (International Group for Reducing IMUP), a not-for-profit organization that now contains more than 140 members of different disciplines from 26 countries. IGRIMUP's goal is to combat IMUP via interdisciplinary communication and collaboration. IGRIMUP has been involved in several international conferences and collaborative research efforts and has published a comprehensive position statement, including action recommendations for policy, research, and education (4). Several other international or continental collaborations have adopted the same goals. However, unlike most epidemics for which specific immunization and treatments are invented quite quickly, and despite the significant burden of IMUP, there is still no general consensus regarding the best methods to combat this insidious problem (1–4). As a start, “immunization” should actually involve prevention through better education of all healthcare providers, health authorities and policymakers about the scale and causes of IMUP and the ways to prevent or mitigate it, which this compendium of articles serves to do.

In reducing IMUP, most geriatricians are familiar with and rely on explicit (criteria-based) tools such as the Beers criteria (11), the START/STOPP (12), the FORTA list (13), or similar country specific medication optimization lists. It is important to note that some lists just point to “bad” drugs to avoid (negative lists such as the Beers criteria), while few also identify potentially omitted “good” drugs to be added to medication (positive-negative lists such as START/STOPP or FORTA). Probably, using the latter approach is clinically more successful than using “drugs-to-avoid” lists; e.g., Wehling et al. (13, 14) demonstrated in the VALFORTA-RCT that adverse drug effects were lowered at a number-needed-to-treat of only 5. However, all these lists and attempts to discriminate “bad drugs” from “good drugs” may be misleading, are insufficiently accurate to use as stand-alone measures of prescribing quality (15) and, in particular, negative lists do not take into consideration specific patient characteristics. Prescribing 15 “non list,” apparently “good medications” to older patients is still likely to do more harm than good (16). Similar to “drugs to avoid” lists, the use of more sophisticated computerized decision-making support systems has resulted in a reduction in the number of medications per patient but only a modest reduction in the risk of death and hospitalization, and with no or only minor improvements in other clinical outcomes or quality of life (QoL).

These overall somewhat disappointing findings may be partly explained by the fact that all these strategies—with variations—do not encompass a whole patient perspective, perhaps because they derive from a guidelines-based worldview based on the single-disease model, which posits that patients are largely homogeneous. Such a paradigm is incongruent with the reality of older multimorbid populations where heterogeneity is the norm and the natural histories of diseases are hard to define, due to the inseparable comingling of multiple diseases with multiple drugs (3, 6).

Evidence is accumulating that apart from prevention i.e., not commencing medications, the most powerful strategy to combat IMUP is deprescribing i.e., the judicious cessation or withdrawal of unnecessary or inappropriate medications (5, 6, 10, 16), and the addition of omitted positively labeled drugs (17). We believe that a patient centered approach is more appropriate in advanced age and particularly in VOCODFLEX. Furthermore, it is important to adopt a palliative approach such as the Holmes' Pyramid (18) which postulates that as life expectancy decreases, the goals of care focus less on disease prevention or active treatment and more on QoL, allowing the scope of appropriate medications to contract substantially.

Each of the co-editors of the present Research Topic has proposed and promoted a deprescribing method, FORTA in Germany (19), the 10 steps in Australia (20, 21), and the GPGP in Israel (5, 6, 10, 16). We base our methods on the hypothesis that, when combining advancing age with a growing comorbidity burden, the clinical and economical harm of certain drugs outweighs the sum total of their beneficial effects, and they should therefore serve as targets for deprescribing.

Things look even more positive if both over- and undertreatment are addressed by proper listing approaches eventually leading to a lower impact of the number of drugs, and a higher impact of the quality of the medication scheme. An 80-year old patient with three–four relevant disease may benefit from 10 to 12 drugs if they are positively labeled and properly indicated.

Thus, in the future, drug optimizing strategies should no longer be only focused on de-prescribing, but on re-prescribing describing the exchange of “bad” by “good” drugs where feasible (17). In other words: we should not aim at only reducing “polypharmacy” or the number of drugs, but on the age-appropriateness of all medications.

In order to achieve a patient centered individualization of drug therapy and in line with the palliative approach, we should incorporate patient/family characteristics and preferences, gain their confidence and elicit their consent to the process of de- or re-prescribing.

There is a fair consensus that five drugs constitutes the cut-off for polypharmacy and that beyond this number, older people experience increased risk of mortality, disability, frailty and falls (22), although IMUP can occur with even a single medication. Still, the level of inappropriate medication use (IMU) does correlate with the number of drugs (Polypharmacy) and it was also proven in two longitudinal studies that, not only does IMUP increase with the number of drugs prescribed but, conversely, the beneficial effects of deprescribing increases with the magnitude of deprescribing (5, 6).

De-and re-prescribing should be regarded as an essential part of good prescribing (17, 23, 24). However, the reluctance of many doctors to adopt de-and re-prescribing remains a significant barrier to realizing its benefits for patients (5, 6, 17, 25) The paucity of evidence-based deprescribing guidelines has meant that even knowledgeable, ethical and well-intentioned clinicians lack the confidence to de-prescribe and thus continue to harm their most vulnerable patients. Fortunately, evidence is growing that closely supervised and well considered de-prescribing is safe and achieves beneficial effects on clinical outcomes and QoL that can appear relatively quickly and be sustained over several years (5, 6). This should encourage doctors to more routinely consider deprescribing medications whenever appropriate.

Applying deprescribing globally could improve the quality of later years of life for millions of older people and also forego the costs of drugs and hospitalizations resulting from IMUP—a triple clinical economical win– win situation.

Nothing short of a revolution in our clinical thinking will suffice to stem the tide of the devastating IMUP epidemic. All clinicians, including specialists, carry responsibility for prescribing too many medications to older patients. Other health professionals, policy makers and the media should join forces in an attempt to increase awareness about IMUP among doctors and the general public, and to promote routine use of deprescribing for older patients.

In recent times, there seems to have developed a greater receptiveness on the part of doctors and patients to IMUP and deprescribing (21, 26, 27). More patients today believe that they are taking too many medications (28, 29) and are open to deprescribing. In a US-population based study, Reeve at al. (26) surveyed the attitudes of older adults toward deprescribing and found that most respondents were willing to have at least one medicine deprescribed and did not report distress surrounding this decision. Certainly, education of patients and care givers is an essential component of our campaign to prevent or reduce IMUP, and education about deprescribing must begin early within the core curricula of medical and pharmacy schools. Improved communication and information around the potential benefits of medication reviews which culminate in de- and re-prescribing may enhance patient engagement and improve their experience and outcomes (30). Furthermore, media stressing the point that deprescribing is an essential part of good medical practice will help change public attitudes and instill awareness that any prescribed drug, originally started because it conferred benefit, may eventually need to be stopped if it subsequently risks causing harm (17, 23, 26).

In conclusion, optimizing medication management in older adults requires a multidisciplinary approach focusing on regular, comprehensive drug reviews to combat polypharmacy, adverse drug effects, and non-adherence. Older adults are at increased risk of medication-related harm, including hospitalizations, because of decreased renal and hepatic clearance, drug-drug and drug-disease interactions due to polypharmacy and multiple co-morbidity burden, and cognitive and physical impairments.

Key strategies include the following:

- Comprehensive medication review: regular, in-depth assessments by healthcare professionals (family doctors, geriatricians, pharmacists) to identify and address medication-related issues, and ensure an accurate, up-to-date medication list is maintained, especially during transitions of care (e.g., hospital to home). This list should reflect both over- and undertreatment issues.
- Individualized care: setting treatment goals that prioritize quality of life, independence and functional capacity over strict, evidence-based treatment targets for every disease.
- De- and re-prescribing: systematically reviewing and withdrawing unnecessary, ineffective, or harmful medications in patients at risk of medication harm because of polypharmacy, frailty, cognitive impairment and living alone, and adding beneficial, but omitted drugs.
- Simplify regimens: reducing the dosing frequency and number of medications to improve adherence.
- Optimize adherence: utilizing pill organizers (Webster packs, dosette boxes, and other dosing aids), apps, and reminders to assist safe and consistent administration of medications.
- Provide carer support: educate family and carers in supervising medication administration, recognizing and managing adverse effects of medication, and supporting their loved ones in the processes of deprescribing.
